# High Uptake of Systematic HIV Counseling and Testing and TB Symptom Screening at a Primary Care Clinic in South Africa

**DOI:** 10.1371/journal.pone.0105428

**Published:** 2014-09-30

**Authors:** Annelies Van Rie, Kate Clouse, Colleen Hanrahan, Katerina Selibas, Ian Sanne, Sharon Williams, Peter Kim, Jean Bassett

**Affiliations:** 1 Department of Epidemiology, University of North Carolina at Chapel Hill, Chapel Hill, North Carolina, United States of America; 2 Clinical HIV Research Unit, University of the Witwatersrand, Johannesburg, South Africa; 3 National Institute of Allergy and Infectious Diseases, National Institutes of Health, Bethesda, Maryland, United States of America; 4 Witkoppen Health and Welfare Center, Johannesburg, South Africa; McGill University Health Centre, McGill University, Canada

## Abstract

**Background:**

Timely diagnosis and treatment of tuberculosis (TB) and HIV is important to reduce morbidity and mortality, and break the cycle of ongoing transmission.

**Methods:**

We performed an implementation research study to develop a model for systematic TB symptom screening and HIV counseling and testing (HCT) for all adult clients at a primary care clinic and prospectively evaluate the 6-month coverage and yield, and 18-month sustainability at a primary care clinic in Johannesburg, South Africa.

**Results:**

During the first 6 months, 26,515 visits occurred among 12,078 adults. The proportion of adults aware of their HIV status was 43.7% at the start of the first visit, increased to 84.6% at the end of the first visit, and to 90% at end of any visit during the first 6 months. During these 6 months, 1042 clients were newly diagnosed with HIV. HIV prevalence was 22.9% among those newly tested, and 58.9% among all adult clinic clients. High coverage of systematic HCT was sustained across all 18 months. Coverage of systematic HIV-stratified TB symptom screening during first 6-months was also high (89.6%) but only 35.0% of those symptomatic were screened by sputum. During these 6-months, 90 clients had a positive Xpert MTB/RIF assay, corresponding to a TB prevalence of 0.4% among all 23,534 clients TB symptom-screened and 2.8% among the 3,284 clients with a positive TB symptom screen. The initial high coverage of TB symptom screening was not sustained, with coverage of TB symptom screening dropping after the first six months to 70% and assessment by sputum dropping to 15%.

**Conclusion:**

Routine, systematic HCT and HIV-stratified TB symptom screening is feasible at primary care level. Systematic HCT doubled the proportion of clients with known HIV status. While HCT was sustainable, coverage of systematic TB screening dropped significantly after the first 6 months of implementation.

## Introduction

The global burden of tuberculosis (TB) and human immunodeficiency virus (HIV) remains enormous. In 2012, there were an estimated 35.3 million people living with HIV (PLWH), 2.3 million new HIV infections, 1.3 million AIDS deaths, 8.6 million new cases of TB, and 1.3 million TB deaths, including 320,000 among PLWH [1], [2]. In sub-Saharan Africa, there is a high prevalence of undiagnosed TB in the community [3]–[5], many people are unaware of their HIV status [6], and people often enter care at advanced stages of disease [7], suggesting that the current public health approach to diagnosis of TB and HIV is insufficient.

HIV counseling and testing (HCT) is predominantly delivered through voluntary counseling and testing or provider-initiated counseling and testing (PICT). Even though the World Health Organization (WHO) recommends PICT for all people visiting a health facility [8], PICT in health care facilities in high burden countries is mainly targeted at individuals presenting for antenatal, TB or STI care [9]. Alternative approaches such as testing contacts of index cases, mobile testing, door-to-door testing, and school-based testing can increase HIV case finding [10]. A similar situation holds for TB, with suboptimal case finding using the conventional approach of passive case finding among people presenting to health facilities with symptoms suggestive of TB [1], [11]. In pursuit of a more active approach to TB case detection, the WHO recommends intensified case finding among PLWH and close contacts of individuals with infectious TB [12], [13]. Mobile TB screening, door-to-door screening, school-based programs, and community sputum collection point programs have been evaluated but are not routinely implemented [4], [14], [15].

Despite high rates of missed TB and HIV case finding at health facilities [16]–[19], few studies have evaluated the impact of systematic TB symptom screening and HCT for all clients, independent of the presence of risk factors. We aimed to develop a primary care model for systematic TB and HIV screening for all adults presenting to a primary care clinic. We evaluated the model's impact on knowledge of HIV status of clinic clients, HIV and TB case finding, and assessed feasibility and sustainability at a primary care clinic in Johannesburg, South Africa.

## Methods

### Ethics statement

The study was approved by the Institutional Review Board of the University of North Carolina at Chapel Hill, USA, and the Human Research Ethics Committee of the University of the Witwatersrand, Johannesburg, South Africa. Clinic clients provided written consent for use of routine clinic data for research purposes.

### Study setting

The prospective implementation research study took place at the Witkoppen Health and Welfare Centre (WHWC), a primary care clinic serving Diepsloot, a densely populated informal settlement community. Diepsloot, located in northern Johannesburg, is the 5^th^ most deprived of the 420 geopolitical subdivisions of Gauteng Province, South Africa [20].

Prior to the intervention, clinic clients were referred for HIV counseling and testing either through self-request (voluntary HCT) or by their provider (PICT) who targeted clients of antenatal care, tuberculosis and sexually transmitted disease services. Symptom screening for the presence of prolonged cough and weight loss was initiated by the care provider at time of clinical evaluation, either systematically among PLWH or as part of targeted clinical assessment in others. Collection of sputum was performed if requested by the nurse or doctor.

### Development of a primary care model for integrated routine screening for TB and HIV

Model development first involved meetings with the clinic leadership to discuss preliminary findings [18], map current patient flow, and identify possibilities for re-allocation of space and distribution of new tasks. Meetings were then held to receive input from clinic staff routinely involved in TB and HIV care and treatment. To establish the final model, an iterative process was implemented over a 12-month period using participatory quality improvement methods. Once the final model was developed, the TB symptom screening and HCT activities became routine clinic activities and were no longer supported or monitored by research staff.

### Data collection

All data were collected as part of routine patient care. Data used in the analysis were retrieved from paper-based medical files and clinic registers, as well as electronic clinic records and laboratory data. Clinic files of all adults (age ≥18 years) visiting the clinic between 30 Jan 2012 and 27 July 2012 were reviewed and data were collected on age, gender, pregnancy status, nationality, employment, HIV status (known positive, negative test result in prior 3 month period, unknown), outcome of HCT (positive, negative, not done), CD4 count if newly diagnosed with HIV, outcome of HIV-stratified TB symptom screening (positive, negative, not done), collection of sputum (if positive symptom screen), and Xpert MTB/RIF result.

To determine long-term sustainability, an additional file review was performed for 162 clients randomly selected among those visiting the clinic between January 28 to February 1, 2013 and 142 clients visiting the clinic during July 29 to August 2, 2013.

### Statistical analysis

Characteristics of clinic clients are described using proportions for categorical variables, and medians and interquartile ranges (IRQ) for continuous variables. Detailed analysis of uptake and results of HCT by prior HCT status was performed for all clinic clients at the time of their first visit to the clinic during the six-month period. To determine changes in weekly HIV and TB screening outcomes of all visits (including repeat visits by some clinic clients), linear regression modeling techniques were used, incorporating an autoregressive error model to correct for the time-series structure and autocorrelation. The model output consists of a slope estimate (β) corresponding to an increase or decrease per week during the 6-month period. For example, a β of 0.001 corresponds to an increase of 0.1% per week. Log-binomial regression modeling was used to identify factors associated with testing outcomes, with results presented as crude risk ratios (RR) and 95% confidence intervals (95%CI).

## Results

### Model development for integrated systematic screening for TB and HIV at primary care

During the iterative, 12-month participatory process, changes were made to leverage existing space and staff, simplify patient flow and reduce waiting times. Conditions agreed upon between investigators and clinic staff were to: (1) integrate activities into current clinic activities instead of creating separate screening officers [21] (2) make HIV counseling part of routine care and perform HIV testing in those with unknown HIV status unless the client refuses, (3) have HCT precede TB screening to allow HIV-stratified TB symptoms screening, and (4) perform HCT and TB symptom screening during the time people wait to see a nurse or clinician so that results are available at time of clinical evaluation. Two TB symptom checklist stamps were developed to guide health care workers in performing a rapid HIV-stratified TB screen. For PLWH, symptoms suggestive of TB were cough, fever, night sweats and loss of weight for any duration [12]. For HIV-negative individuals, prolonged (≥2 weeks) cough or fever, night sweats and substantial weight loss were considered suggestive of active TB.

The final model involved staff across the spectrum of clinic personnel. The administrative staff registering clinic clients at presentation reviewed the client's file to determine the individual's need for HCT. People who visited WHWC for the first time and returning clients without documented HIV-infected status or a documented HIV negative test performed more than three months prior were judged in need of HCT and referred for group HIV pretest counseling. Lay counselors performed HIV pretest counseling in small groups throughout the morning prior to a client's clinical evaluation. At the end of the group session, people were invited for one-on-one rapid HIV testing and post-test counseling in a private setting. Upon completion of post-test counseling, the counselor placed the appropriate TB symptom screen stamp in the patient's clinic file. It was then the task of the clinic staff performing the routine assessment of vitals (weight, temperature, blood pressure and pregnancy test) to complete the information in the TB symptom screen stamp and to follow the simple algorithm to determine which individuals had a positive TB symptom screen. The same staff decided whether collection of a sputum sample was indicated. Between July 1, 2011 and July 31, 2012, Xpert MTB/RIF assay was performed at point-of-care, with the goal to have the result ready by the time the patient was seen by the nurse or doctor. After August 1, 2012, sputum samples were transported to a centralized laboratory for analysis. If the first-line clinic staff did not request a sputum sample, the nurse of doctor could still request this upon completion of the history-taking and clinical exam.

### Clinic population

A total of 26,515 visits by 12,078 adults occurred at the clinic during the 6-month period (30 Jan 2012 to 27 July 2012) after the model was finalized as described above. The majority (69.5%) of clinic clients were women, 16.8% of whom were pregnant (Table 1). Ages of clients spanned the entire adult range, with a median age of 35 years (IQR 28-43), and 13.7% being 50 years or older. Almost one in three (32.3%) were not of South African nationality, and half (51.7%) were unemployed.

**Table 1:** Characteristics of clients seeking care at a primary care clinic in Johannesburg, South Africa.

	Overall	Male	Female
Number of clinic visits	26515	7831	18684
Number of individual clinic clients, n (%)	12078	3687 (30.5)	8391 (69.5)
Number of visits per client, median (range)	2 (1–11)	2 (1–11)	2 (1–10)
Age at first visit			
Median (IQR)	35 (28–43)	36 (30–44)	34 (27–43)
18–29 years	3799 (31.5)	920 (24.2)	2879 (34.3)
30–39 years	4221 (35.0)	1375 (37.3)	2846 (33.9)
40–49 years	2407 (19.9)	876 (23.8)	1531 (18.3)
≥50 years	1651 (13.7)	516 (14.0)	1135 (13.5)
Pregnancy status at first visit, n (%)*			
Pregnant	—	—	1360 (16.8)
Not pregnant	—	—	6729 (83.2)
Nationality, n (%)#			
Born in South Africa	8172 (67.8)	2395 (65.1)	5777 (68.9)
Born outside of South Africa	3890 (32.3)	1285 (34.9)	2605 (31.1)
Employment status at first visit, n (%)**			
Employed	5827 (48.3)	1966 (53.4)	3861 (46.0)
Not employed	6246 (51.7)	1717 (46.6)	4529 (54.0)

IQR: interquartile range;

* pregnancy status missing in 302 (3.6%) of women;

# nationality missing in 16 (0.1%) individuals;

** employment status missing in 5 (0.0) individuals.

### Uptake of HIV counseling and testing

At time of arrival for the first clinic visit during the 6-month period, 41.2% (95%CI: 40.3–42.0%) of the 12,078 adults were known HIV positive, 2.5% (95%CI: 2.2–2.8%) had a documented HIV negative test within the past 3 months, and 54.7% (95%CI: 53.8–55.6%) had an unknown HIV status (Table 2). Of those with unknown HIV status, the vast majority (88.2%) had never been tested for HIV at WHWC. Of those with unknown status, 74.8% (95%CI: 73.6–75.9%) were tested during their first clinic visit. The proportion tested was higher for those never previously tested at WHWC (77.9%, 95% CI 76.8–79.0%) compared to those tested more than 3 months ago (51.7%, 95% CI 48.2–55.2%). All except one person with a prior negative HIV test had a repeat negative HIV test. Among the 4537 tested for the first time at the clinic, 1041 new cases of HIV were detected (22.9%, 95% CI 21.7–24.2%). The number needed to test to identify one new case of HIV was 4.7 among all 4940 clients tested and 6.3 among all 6603 clients who received pretest counseling. By the end of the first clinic visit during the 6-month period, 84.6% (95% CI 83.9–85.2%) of all adult clients were aware of their HIV status, of which 58.9% were HIV positive. Failure to receive a documented HIV status during the first clinic visit was not associated with gender (RR 0.97, 95%CI: 0.93–1.02) but was higher among younger clients (age<40 years, RR 1.25, 95% CI 1.19–1.30) and those with foreign nationality (RR 1.10, 95% CI 1.05–1.15).

**Table 2:** Uptake and results of HIV counseling and testing among 12,078 individuals visiting a primary care clinic in Johannesburg, South Africa.

	Overall		Male		Female	
	N*	%*	N*	%*	N*	%*
**HIV status at start of first visit**						
Known positive	4972	41.2	1481	40.2	3491	41.6
Known negative (test ≤3 m)	304	2.5	51	1.4	253	3.0
Unknown	6603	54.7	2103	57.0	4500	53.6
Prior tested (>3 m)	779	11.8	134	6.4	645	14.3
Not tested previously	5824	88.2	1969	93.6	3855	85.7
Clinic file not found	199	1.7	52	1.4	147	1.8
**Outcome of HCT** (among those with unknown HIV status)						
Not tested	1663	25.2	592	28.2	1071	23.8
Tested	4940	74.8	1511	71.9	3429	76.2
All tested						
HIV positive	1042	21.1	354	23.4	688	20.1
HIV negative	3898	78.9	1157	76.6	2741	79.9
Prior test (>3 m) negative						
Tested	403	51.7	58	43.3	345	53.5
HIV positive	1	0.2	0	0.0	1	0.3
HIV negative	402	99.8	58	100.0	344	99.7
Not tested previously						
Tested	4537	77.9	1453	73.8	3084	80.0
HIV positive	1041	22.9	354	24.4	687	22.3
HIV negative	3496	77.1	1099	75.6	2397	77.7
Initial CD4 count if newly diagnosed with HIV						
Median (IQR) cells/µl	268	136–412	221	94–352	295	169–427
<50 cells/µl	87	8.3	46	13.0	41	6.0
50–199 cells/µl	262	25.1	109	30.8	153	22.2
200–350 cells/µl	283	27.2	92	26.0	191	27.8
>350 cells/µl	330	31.7	86	24.3	244	35.4
Missing	80	7.7	21	5.9	59	8.6
**HIV status at end of clinic visit**						
Known	10216	84.6	3043	82.5	7173	85.5
Known positive	6014	58.9	1835	60.3	4179	58.3
Known negative	4202	41.1	1208	39.7	2994	41.7
Unknown	1862	15.4	644	17.5	1218	14.5

* N and % unless indicated otherwise the estimated proportion.

**Figure 1:**
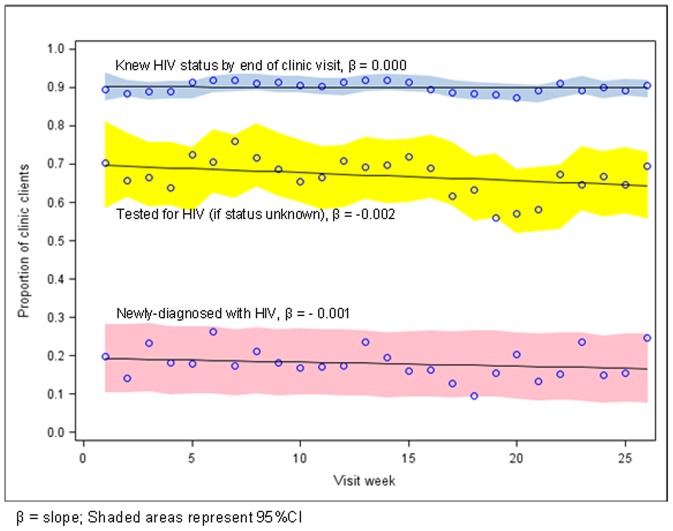
Systematic HIV counseling and testing at primary care clinic in Johannesburg, South Africa: coverage, case detection and sustainability over a 26-week period.

To assess sustainability, we assessed changes over time in the proportion of clients knowledgeable of their HIV status by the end of *any* clinic visit (i.e. first and repeat visits) (Figure 1). During the 6-month period, 90.0% (95%CI: 89.7–90.4%) of clients were aware of their HIV status by the end of any visit. This proportion did not change during the 6-month period (β = 0.000) and remained similar 6 and 12 months later (92.6%, p = 0.27 and 86.0%, p = 0.12), suggesting long-term sustainability of routine HCT.

While the HIV prevalence among newly-tested clients at *any* visit decreased from an estimated 19.5% (95%CI: 10.5–28.3) to 16.4% (95%CI: 7.5–25.3) during the 6-month period (Figure 1), HIV prevalence among clients newly tested when visiting the clinic for the *first* time was 22.9% and increased from an estimated 19.5% (95%CI: 10.5–28.3) to 23.1% (95% CI: 11.4%–34.8%).

The median CD4 value at the time of a new HIV diagnosis was 260 cells/µl (IQR 136–412). Newly-diagnosed men had more advanced disease compared to women (221 CD4 cells/µl, IQR: 94–352 among men vs. 295 cells/µl, IQR: 169–427 among women, p<0.001).

### Uptake of HIV-stratified TB symptom screening

**Figure 2:**
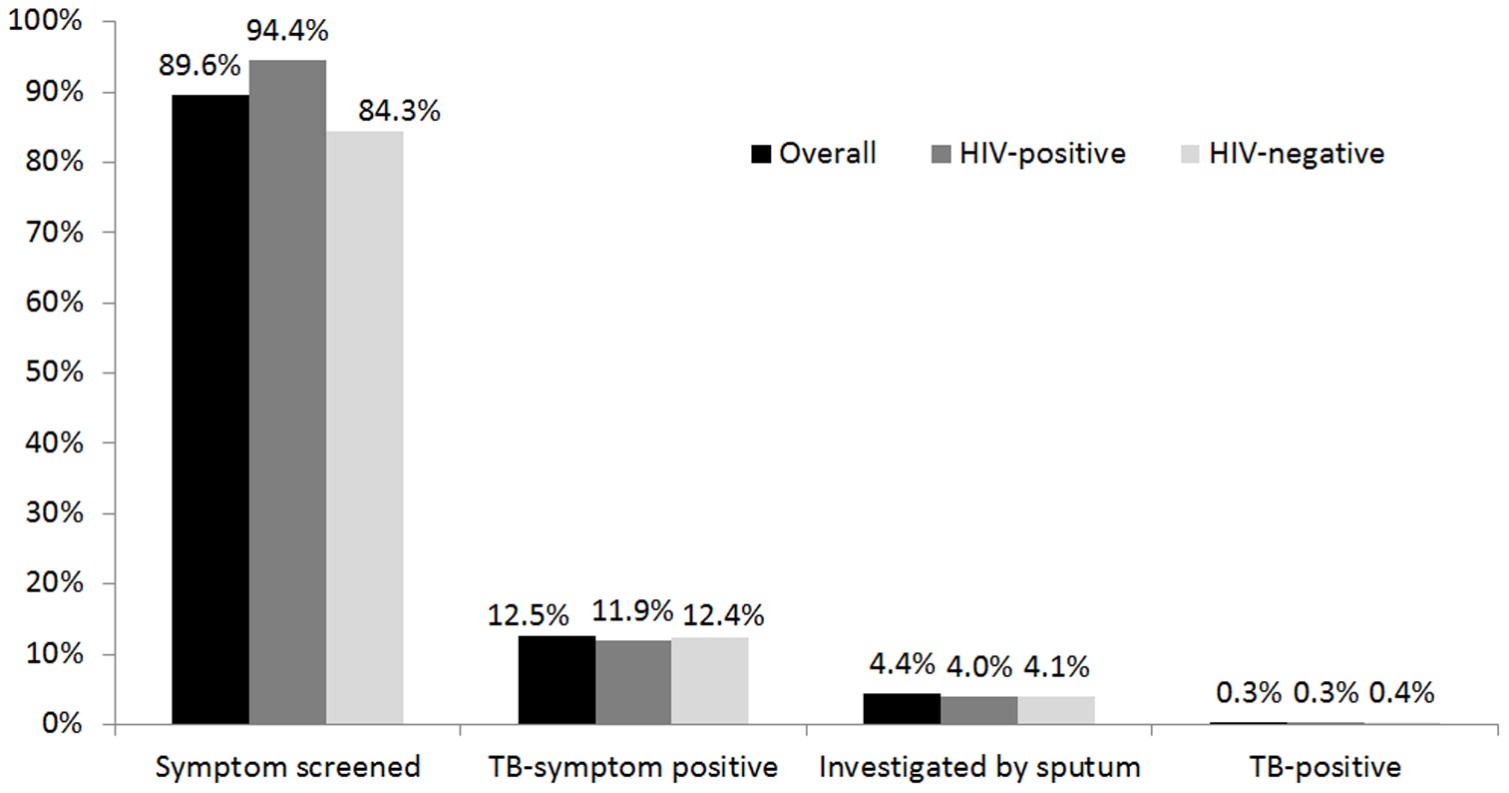
The TB screening cascade observed at 26,279 clinic visits at a primary care clinic in Johannesburg, South Africa. Black bars represent screenings in all clinic clients, dark grey bars represent screenings in HIV-positive clinic clients, and light grey bars represent screenings in HIV-negative clinic clients.

Information on completion of a TB symptom screen was available for 26,279 clinic visits during the 6-month period (Table 3). A TB symptom screen was performed at 89.6% (95% CI 89.2–89.9%) of the visits (Figure 2). Of the 23,534 symptom screens performed, 14.0% (95% CI 13.5–14.4%) were positive. Among the 3284 visits with a positive TB symptom screen, a sputum specimen was collected in 1150 (35.0%, 95%CI: 33.4–36.7%), of which 90 (8.0%, 95% CI 6.5–9.7%) were positive for *Mycobacterium tuberculosis* on Xpert MTB/RIF. Overall, 90 cases of TB were detected, corresponding to a 7.8% case detection among the 1150 cases screened by sputum, 2.7% case detection rate among the 3284 clients with a positive symptom screen and 0.4% case detection among all 23534 clients screened for TB symptoms.

**Table 3:** Uptake and results of TB symptom screening and sputum testing during 26,279 clinic visits at a primary care facility in Johannesburg, South Africa.

	All visits		Male		Female		HIV-positive		HIV-negative		HIV-unknown	
	N	%	N	%	N	%	N	%	N	%	N	%
Screened for TB symptoms at visit*												
No	2745	10.5	803	10.3	1942	10.5	952	5.6	1023	15.7	770	29.4
Yes	23534	89.6	6962	89.7	16572	89.5	16181	94.4	5502	84.3	1851	70.6
Presence of TB symptoms among those screened												
No	20250	86.1	5778	83.0	14572	87.3	14145	87.4	4690	85.2	1415	76.5
Yes	3284	14.0	1184	17.0	2100	12.7	2036	12.6	812	14.8	436	23.5
Investigation by sputum among those with positive symptom screen												
No	2134	65.0	726	61.3	1408	67.0	1355	66.5	547	67.4	232	53.2
Yes	1150	35.0	458	38.7	692	33.0	681	33.5	265	32.6	204	46.8
Xpert result among those investigated by sputum**												
Negative	1030	89.8	393	86.0	637	92.3	609	89.7	238	89.8	183	90.2
Positive	90	7.9	53	11.6	37	5.4	51	7.5	23	8.7	16	7.9
Error or invalid	27	2.4	11	2.4	16	2.3	19	2.8	4	1.5	4	2.0

* Clinic file could not be located for 229 patients;

** Excludes 3 missing Xpert results.

There was no difference in TB symptom screening by gender (RR 1.00. 95%CI: 0.99–1.01), but men were more likely to be investigated by sputum (RR 1.45, 95%CI: 1.31–1.62) and were twice as likely to test positive for *M. tuberculosis* (RR 2.24, 95%CI: 1.53–3.26). HIV-positive clients were more likely to be screened for TB symptoms (94.4%) compared to HIV-negative clients (84.3%); those with unknown HIV status were least likely to be screened (70.6%). Case detection rate was similar among the three groups (7.5%, 8.7% and 7.9% for HIV-positive, HIV-negative and HIV-unknown, respectively). The number of clients needed to symptom screen to identify one case of TB was 262 overall, 131 for male clients, 448 for female clients, 317 for HIV infected clients, 239 for HIV uninfected clients and 116 for clients with unknown HIV status.

**Figure 3:**
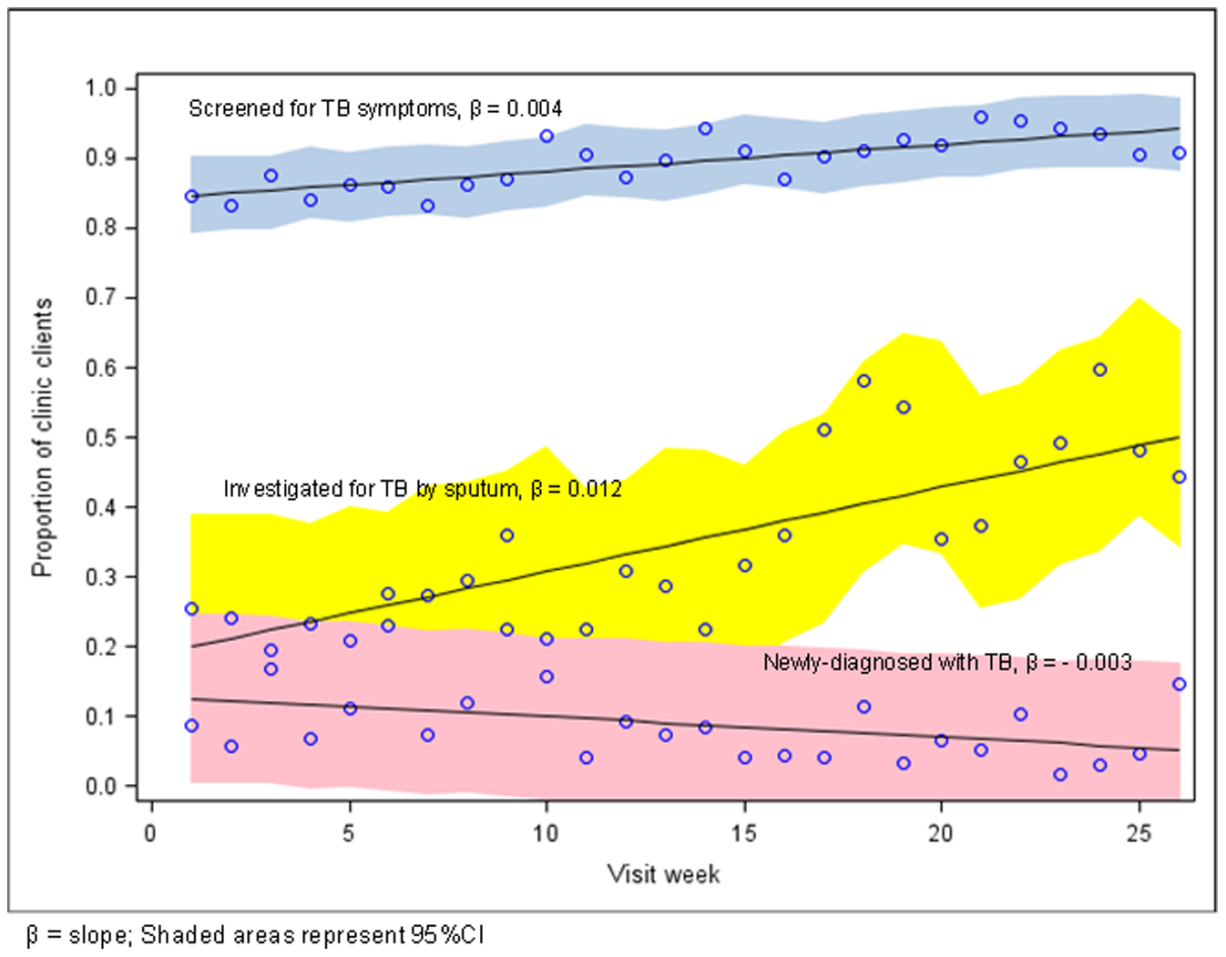
Systematic TB symptom screening and assessment at primary care clinic in Johannesburg, South Africa: coverage, case detection, and sustainability over a 26-week period.

During the 6-month period, an increase over time was observed for the uptake of TB symptom screening (from an estimated 84.7 (95%CI: 79.1–90.3%) to 93.5% (95%CI: 88.2–98.7%), β = 0.004) and the proportion of TB suspects investigated by sputum (from an estimated 19.9% (95%CI: 9.5–38.9%) to 49.8% (95%CI: 34.1–65.4%), β = 0.012) (Figure 3). Associated with the increase in the proportion of clients assessed by sputum, the proportion Xpert MTB/RIF positive among those assessed by sputum decreased (β = −0.003) from an estimated 12.6% (95%CI: 4.3–24.7%) to 5.3% (95%CI: 0.0–17.5%).

TB screening coverage was not sustained long-term, with significantly lower proportion of clients screened for TB symptoms 6 and 12 months later (68.5% and 71.0%, respectively, p<0.001), and significantly lower proportion of TB suspects assessed by sputum 6 and 12 months later (15.3% and 13.6%, respectively, p = 0.090).

## Discussion

In this study, the first to our knowledge to implement and evaluate a primary care model for integrated systematic HIV counseling and testing and TB symptom screening, we achieved high rates of TB symptom screening and HCT when implemented for all adult clients at a primary care clinic in South Africa, with 89.6% being screened for TB symptoms and 84.6% of people being aware of their HIV status by the end of their first clinic visit. Six months of systematic screening for HIV and TB resulted in the detection of 1042 cases of HIV, corresponding to a 22% HIV prevalence among those with unknown status, and 90 new cases of TB, corresponding to a 2.7% TB prevalence rate among those with a positive symptom screen.

Missed opportunities for HIV testing at health facilities are common, and only testing those perceived to be at high risk of HIV can miss large numbers of PLWH [19]. Implementation of routine HCT doubled the proportion of individuals knowledgeable about their HIV status, from 43.7% at the start of the clients' first clinic visit to 84.6% by the end of the clinic visit. HCT uptake was higher than that reported for other facility-based HCT approaches, and similar to the 80–85% uptake achieved in community-based HCT programs [10].

HIV prevalence among those with unknown HIV status who accepted testing was 19.5%, slightly higher but not statistically different from the 16.1% estimated population HIV prevalence in Gauteng Province in 2011 [22]. This is in contrast with results of a meta-analysis, which found that the HIV positivity rate at facility-based HCT was, on average, double that of community-based HCT [10]. Despite high uptake, HCT did not result in high proportions of diagnoses at early stages of disease, with only 31.7% diagnosed at CD4 count >350 cells/µl, substantially lower than the 40.2% estimate in a meta-analysis of individuals diagnosed with CD4 count >350 through community-based HCT [10]. Taken together, these data suggest that systematic HIV testing at facility levels can reach similar case finding rates as community-based HCT but captures individuals at more advanced level of disease progression.

Reports of missed opportunities for TB case finding at primary care clinics suggest that TB case finding should not be limited to passive case finding or PLWH [17]. We achieved high (89.6%) coverage of HIV-stratified TB symptom screening of all clinic clients, suggesting operational feasibility under programmatic conditions. The proportion of individuals with positive symptom screen assessed by sputum (35%) was lower than expected, in part due to the frequent occurrence of unproductive cough. This is similar to findings at primary care clinics in Botswana, where only 35.3% of those with a positive symptom screen were referred for sputum smear microscopy or X-ray [23]. The TB case finding yield was relatively low, with 90 cases detected, representing 2.8% of the 3,284 clients with a positive TB symptom screen and 0.4% of the 23,534 clients screened. Operational feasibility but low yield of systematic TB symptom screening was also observed in other studies. In Botswana, 926 (8%) of 11,799 clinic clients had a positive symptom screen, 327 (35.3%) were referred for examinations (sputum smear microscopy or X-ray), and 19 (13.4% of those tested, 0.2% of those screened) were diagnosed with TB [23]. In outpatient departments of 32 hospitals in Swaziland [11], 14,998 (6%) of 251,867 clients screened positive and were tested using Xpert, 1499 (10% of those tested, 0.6% of those screened) were diagnosed with TB. In Afghanistan, 22,228 (2.5%) of 889,120 clinic attendees systematically screened for TB at 47 health facilities were tested with smear microscopy of whom 1986 were diagnosed with TB (8.9% of those tested, 0.2% of those screened) [11].

In contrast to our findings of sustainability of systematic HCT, the high coverage of systematic TB screening was not sustained long term. The proportion of individuals symptom screened dropped from 89.6% to about 70% and the proportion with positive symptom screen assessed by sputum dropped from 35% to about 15%. Several factors may have contributed to this observation. First, whereas systematic HIV testing is part of a South Africa national testing campaign, systematic TB symptom screening in all clinic clients is not recommended by the South African Department of Health. Second, the number needed to screen to detect one case is much higher for TB than HIV (262 versus 6), possibly resulting in a perception of higher effectiveness and thus stronger motivation for the health care workers to test for HIV compared to TB. Third, while Xpert MTB/RIF was performed at point-of-care during the study period, samples were sent to a centralized laboratory after the first 6 months. Lack of immediate feedback on sputum results may further have decreased the motivation of the clinic staff to collect sputum for evaluation.

The large sample size, pragmatic approach and the evaluation under real-world conditions using non-research clinic staff to perform all activities are important strengths of our study. The interpretation of the findings however needs to take some limitations into account. The study was a single-site observational study without a control group. The impact of the intervention on HIV and TB diagnoses could therefore not be estimated as the proportion of new HIV and TB diagnoses that would have been made under standard of care was not known. We also do not know if missed diagnoses occurred among those who were not tested for HIV, those with a negative TB symptom screen, and clients with a positive symptom screen who were not assessed by sputum. Inclusion of a historical control using routine clinic statistics could provide some insights but would have inherent limitations due to differences in time periods assessed and use of different data collection standards. Finally, the available data did not allow us to estimate cost effectiveness of the program, or to assess the impact of the intervention on individual morbidity and mortality or transmission of TB and HIV at population level.

## Conclusion

To date, efforts to curb the TB/HIV epidemic have focused on TB screening for PLWH and HCT for people diagnosed with active TB. While outreach screening activities have been shown to be effective, these programs require infrastructure development, complicate linkage to care, and sustainability under programmatic conditions of such programs can be hard to obtain. Improving TB and HIV case finding at health facilities may be a more affordable and sustainable first step to increase case finding in high burden settings. Key features likely contributing to our program's success were full integration of TB symptom screening and HCT into routine clinic activities, distribution of tasks across a range of clinic personnel, and timing of HCT and TB symptom screening during the time clinic clients waited to see a nurse or physician. Future research is needed to explore the epidemiological and contextual factors that improve the effectiveness of different tools and approaches to systematic TB screening at primary care.

## Acknowledgments

We are deeply grateful to all patients and staff at Witkoppen Health and Welfare Center. We would also like to thank members of the research team: Veronica Modise, Bridgette Moatlhodi, and Violet Molepo. We thank Pierre Barker and Michèle Youngleson of the Institute for Healthcare Improvement for guidance and support regarding the use of quality improvement methods.
